# Immune Stimulation Using a Gut Microbe-Based Immunotherapy Reduces Disease Pathology and Improves Barrier Function in Ulcerative Colitis

**DOI:** 10.3389/fimmu.2018.02211

**Published:** 2018-09-27

**Authors:** Ho Pan Sham, Mark Bazett, Momir Bosiljcic, Hyungjun Yang, Beryl Luk, Hong T. Law, Vijay Morampudi, Hong B. Yu, Jim Pankovich, Simon Sutcliffe, Brian Bressler, John K. Marshall, Richard N. Fedorak, Jenny Chen, Michelle Jones, Hal Gunn, Shirin Kalyan, Bruce A. Vallance

**Affiliations:** ^1^Qu Biologics Inc., Vancouver, BC, Canada; ^2^Division of Gastroenterology, Department of Pediatrics, BC Children's Hospital Research Institute (BCCHRI), University of British Columbia, Vancouver, BC, Canada; ^3^Division of Gastroenterology, Department of Medicine, University of British Columbia, Vancouver, BC, Canada; ^4^Department of Medicine and Farncombe Family Digestive Health Research Institute, McMaster University, Hamilton, ON, Canada; ^5^Division of Gastroenterology, University of Alberta, Edmonton, AB, Canada; ^6^Division of Endocrinology, Department of Medicine, University of British Columbia, Vancouver, BC, Canada

**Keywords:** ulcerative colitis, immunotherapy, microbial therapy, inflammation, mucosal immunity, barrier function

## Abstract

**Background:** Current ulcerative colitis (UC) treatments are focused on symptom management primarily via immune suppression. Despite the current arsenal of immunosuppressant treatments, the majority of patients with UC still experience disease progression. Importantly, aggressive long-term inhibition of immune function comes with consequent risk, such as serious infections and malignancy. There is thus a recognized need for new, safe and effective treatment strategies for people living with UC that work upstream of managing the symptoms of the disease. The objective of this study was to evaluate a microbial-based treatment, QBECO, that functions to productively activate rather than suppress mucosal immune function as a novel approach to treat UC.

**Methods:** Two established models of experimental colitis, namely chemically-induced DSS colitis and the spontaneous colitis that develops in *Muc2* deficient mice, were used to assess whether QBECO treatment could ameliorate gastrointestinal disease. A small exploratory 16-week QBECO open-label trial was subsequently conducted to test the safety and tolerability of this approach and also to determine whether similar improvements in clinical disease and histopathology could be demonstrated in patients with moderate-to-severe UC.

**Results:** QBECO treatment successfully reduced inflammation and promoted mucosal and histological healing in both experimental models and in UC patients. The preclinical models of colitis showed that QBECO ameliorated mucosal pathology, in part by reducing inflammatory cell infiltration, primarily that induced by neutrophils and inflammatory T cells. The most rapid and noticeable change observed in QBECO treated UC patients was a marked reduction in rectal bleeding.

**Conclusion:** Collectively, this work demonstrates for the first time that strategically activating immune function rather than suppressing it, not only does not worsen colitis induced-damage, but may lead to an objective reduction in UC disease pathology.

## Introduction

Ulcerative colitis (UC) is a chronic, life-long disorder of the gastrointestinal tract characterized by mucosal inflammation localized solely to the colon. The disease usually develops in adults in their third or fourth decade, and without treatment the uncontrolled inflammation can affect the entire large bowel and lead to an increased risk of colon cancer. Current treatment strategies include mesalamine, glucocorticoids, thiopurines, and biologic agents, all acting to control disease through immune suppression. Unfortunately, none of the immunosuppressive therapies used to date have led to sustained remission for the majority of UC patients. Collectively, only 20–35% of the UC population have completely controlled disease whereas an estimated 40–55% of patients do not respond to current therapies (1). Importantly, long-term chronic use of immunosuppressive and anti-inflammatory drugs comes with an increased risk of infections and certain malignancies (1–4). Taken together, there is clearly a significant unmet need in UC for new therapeutic approaches that provide better health outcomes for patients.

While the precise etiology of UC remains elusive, impaired gut barrier function, as evidenced by increased barrier permeability and reduced thickness of the colonic mucus layer, is considered to be a major contributor to disease pathophysiology, as it allows increased penetration of luminal bacteria into gut tissues (5, 6). It is of note that early life experience to microbes, as assessed by a history of acute infections, has epidemiologically been associated with reduced risk of inflammatory bowel diseases (7); an observation that supports the “hygiene hypothesis” (8). Experimentally, it has been shown that early microbial exposure helps train the immune system to respond appropriately to commensals and maintain an optimal microbial distance at mucosal barrier sites (9). Defects in barrier function in UC are thought to lead to an over-reactive immune response to luminal antigens and a subsequent loss of mucosal homeostasis (10). The role of impaired barrier function in the development of UC is recapitulated in mice lacking the mucin *Muc2*, the major component of the protective gastrointestinal mucus layer (11). At 3 months of age, *Muc2* deficient mice spontaneously present with colitis that progresses over the following months and mimics several aspects of the human disease (11). Thus, a treatment that can limit damage to gut barrier function and achieve mucosal immune homeostasis by retraining the immune response would be a significant advance for restoring bowel integrity for patients living with UC.

An investigational microbe-based immunotherapy, called QBECO, which is formulated from an inactivated strain of a gut pathogen and administered subcutaneously, was found to improve clinical symptoms of inflammatory bowel disease (IBD) in a compassionate use study (12). Instead of suppressing immune function, QBECO treatment is thought to overcome gastrointestinal immune dysregulation by optimizing aberrant or ineffective immune responses by new immune cell production and mobilization. A similar approach using QBKPN, a lung pathogen-based therapy that targets immune-related disorders of the lung, was found to improve disease in asthma, COPD, and lung cancer models (13–15). In the present study we aimed to characterize the effects of QBECO treatment on gastrointestinal pathology using two different experimental models of colitis, the commonly used DSS model of chemical-induced colitis and the spontaneous colitis that develops in *Muc2* deficient (*Muc2*^−/−^) mice. These preclinical studies were followed by an open-label trial in patients with moderate-to-severe UC to assess tolerability and safety with 16 weeks of QBECO treatment, and to obtain objective measures of clinical, endoscopic and histological changes during the study.

## Materials and methods

### QBECO treatment

QBECO is an inactivated pathogenic clinical isolate of *E. coli*, suspended in physiological saline with 0.4% phenol as a preservative. QBECO was supplied by Qu Biologics (Vancouver, BC). For preclinical studies, treatment was administered subcutaneously every second day at rotating sites, which included the abdomen, anterior and lateral thigh. Pilot studies performed by Qu Biologics have shown subcutaneous administration, relative to oral or intravenous, has the greatest efficacy with the most favorable safety profile. For the clinical trial, study subjects were trained to self-administer QBECO subcutaneously every second day for 16 weeks at one of three randomized doses (0.02, 0.05, or 0.10 mL) that were formulated at a standardized predetermined optical density measure. The study dose range was based on a previous trial in patients with IBD for which patients had adjusted their dose based on the presence of a local skin reaction (indicating immune activation). If a local skin response >8 cm was observed, patients reduced the next dose by one level. Treatment compliance was assessed using patients study diaries.

### Animals

C57Bl/6 mice (female, 8–10 weeks old) were purchased from Charles River laboratories (Wilmington, MA) or Envigo (Huntingdon, UK). *Muc2*^−/−^ mice on a C57BL/6 background were bred in the BCCHRI animal facility. All mice were housed in specific pathogen-free conditions on a light-dark cycle with light from 07:00 to 20:00 h at a temperature of 25°C. Mice were fed a standard diet and were provided water *ad libitum*. All experiments were performed according to protocols (A15-0211, AUP-IBD-2017) in direct accordance with guidelines drafted by the Canadian Council on the Use of Laboratory Animals.

### QBECO treatment in animal models

For the treatment of experimental animals, 30 μL of QBECO or a placebo vehicle control (physiological saline containing 0.4% phenol) were administered subcutaneously every second day. Injections were alternated between the lower right abdomen, the lower left abdomen, the upper right chest, and the upper left chest, rotating clockwise for each injection day. *Muc-2*^−/−^ mice were injected subcutaneously with placebo or QBECO every other day for 30 days starting at 3 months of age; spontaneous colitis is normally overt at 4 months of age. For dextran sodium sulfate (DSS) induced colitis, QBECO was given every other day beginning at 10 days prior to DSS administration for the duration of the QBECO treatment period.

### Dextran sodium sulfate (dss)-induced colitis

Experimental colitis was induced by adding 2 or 2.5% (w/v) DSS (MP Biomedical, Santa Ana, California, USA) into the drinking water *ad libitum*. Mice were treated with DSS for 7 days on DSS. For recovery studies, DSS was replaced by water until the day of euthanization. To assess clinical disease activity score body weight, occult blood or the presence of gross blood per rectum, and stool consistency were determined daily as described previously (16, 17). Weight loss <1% counted as 0 points, <5% count as one point, 5–10% as two points, 10–20% as 3 points and more than 20% as 4 points. If animals lose more than 20% weight for more than 3 days and weight do not recover, euthanize the animal. For stool consistency, 0 points were given for well formed pellets; 2 points for pasty and semi-formed stools, which did not stick to the anus, and 4 points for liquid stools that did stick to the anus. Rectal bleed, 0 points for no blood, 2 points for clear redness in stool and 4 points for rectal prolapse. The average of these three scores (body weight, stool consistency, and rectal bleed) gave an overall clinical score.

### Cell preparation and flow cytometry

Single Cell suspension was prepared from spleen by forcing through a 40 μm filter. Erythrocytes in spleen were lysed with RBC Lysis Buffer (Thermo Fisher Scientific, Waltham, Massachusetts, USA) before proceeding to staining. Cells were stained with CD45 (30-F11), CD4 (Gk1.5), CD11c (N418), F4/80 (BM8), CD19 (B4), CD8 (53-6.7), Ly6G (1AB) (BioLegend, California, USA), and CD3 (17A2) (Thermo Fisher Scientific, Massachusetts, USA). Data were acquired on a Cytoflex (Beckman-Coulter, Indianapolis, IN, USA) and analyzed using FlowJo software (version 10.5.0, Ashland, OR, USA).

### FITC intestinal permeability assay

The assay was performed as previously described (18). At Day 7 following DSS exposure, mice were orally gavaged with 150 μl of 80 mg/ml of FITC-dextran (Sigma-Aldrich, St. Louis, Missouri, USA) in PBS 4 h prior to euthanization. Blood was collected by cardiac punctures and collected into STT tubes (BD Bioscience, Franklin Lakes, New Jersey, USA). Plasma was collected and fluorescence from FITC was quantified using a florescent plate reader.

### RNA extraction and quantitative real-time PCR from biopsy samples and mouse tissues

Human biopsy samples were collected and stored in RNAlater (Thermo Fisher Scientific, Waltham, Massachusetts, USA) following endoscopy. Murine tissues were collected and stored in RNAlater immediately following euthanization. RNA was extracted (using the Life Technology RNA extraction kit) followed by cDNA synthesis using iScript cDNA Synthesis (Bio-Rad Laboratories, Hercules, California, USA). Quantitative real-time PCR was subsequently carried out using the Applied Biosystem StepOnePlus PCR system with Applied Biosystem Taqman polymerase (Applied Biosystems, Foster City, California, USA). The following genes were assessed: *CXCL8, IL6, IFNG, TNFA, IL17A, IL18, IL22*, and *DEFB3*. Quantification was performed using the Applied Biosystems software where PCR efficiency for each primer set was incorporated into the final calculations.

### Cytokine measurement in serum and tissues

Tissue homogenate or serum collected at the time points specified had their cytokine and chemokine levels measured by ELISA kits according to the manufacturer's instructions.

### Immunofluorescence staining

Immunofluorescence staining of control and colitic tissues was performed using previously described procedures. In brief, paraffin embedded tissues were cut (6 μm) and deparaffinized, blocked and stained with primary antibodies. The primary antibodies used targeted Ly6G (Thermo Fisher Scientific, Waltham, Massachusetts, USA) or CD3 (Abcam, Cambridge, United Kingdom) while the secondary antibody was AlexaFluor 568-conjugated goat anti-rabbit IgG. Tissues were mounted using ProLong Gold Antifade reagent (Life Technologies, Carlsbad, California, USA) containing DAPI for DNA staining. Sections were viewed on a Zeiss AxioImager microscope and images taken using an AxioCam HRm camera operating through AxioVision software (Carl Zeiss AG, Oberkochen, Germany).

### Quantification of Ly6G, CD3, or reg3β positive cells

Tissues were stained with anti-Ly6G (Becton Dickinson, New Jersey, USA), anti-CD3 (Abcam, Cambridge, United Kingdom) or anti-Reg3β (R&D Systems, Minneapolis, Minnesota, USA) antibodies and mounted with ProLong Gold Antifade with DAPI (Life Technologies, Carlsbad, California, USA). Pictures were then taken for each tissue section and positively stained cells counted from at least 4 tissues section.

### Clinical trial design

A Phase 2, open-label study (NCT02426372) for the treatment of moderate to severe UC (defined by a Mayo score of between 6 and 12 at screening) was conducted. The Mayo score is a composite of 4 sub-scores: stool frequency, rectal bleeding, endoscopic findings, and physician's global assessment. The original study was intended to assess both the induction (Week 16) and maintenance (Week 52) of clinical remission in 40 patients with UC treated with QBECO. Due to unexpected delays in GMP production of QBECO and consequent trial design changes, both the duration of study (16 weeks) and number of patients enrolled and treated (*n* = 11) were reduced.

The primary objectives of the trial were: assessment of safety (adverse events, clinical laboratory findings, concomitant therapies) and tolerability. The secondary objectives were: clinical response at Week 16, induction of clinical remission at Week 16, and endoscopic healing at Week 16. Exploratory analysis included colonic histological assessment, colonic tissue neutrophil assessment, blood cytokine levels, and colonic tissue gene transcription.

All study patients provided written informed consent, and the trial was conducted within the tenets of the Declaration of Helsinki and Good Clinical Practice guidelines. The study protocol was approved by Health Canada (Control #162034) and the institutional research ethic review board at each of the three study sites.

### Study subjects

Individuals 18 years of age or older, with a diagnosis of UC for at least 6 months, as established by endoscopic and clinical assessment, were eligible to enroll in the study. Patients had to have active moderate to severe UC as defined by a cumulative Mayo score of 6–12 at screening consisting of: an endoscopic sub-score of ≥2 indicative of active disease; a rectal bleeding sub-score of ≥1; and a physician's global assessment sub-score of ≥2. Continued use of stable doses of the following medications were allowed: ≤4.8 g/day of oral 5-ASA, ≤30 mg/day oral prednisone, ≤9 mg/day oral budesonide, and 6-mercaptopurine, azathioprine and methotrexate, providing their dose had not been changed within 4 weeks prior to baseline endoscopy.

Exclusion criteria included the use of TNF-α blockers or vedolizumab within 60 days, intravenous corticosteroid within 2 weeks, rectal 5-ASA or rectal corticosteroids within 2 weeks, experimental or investigation therapies within 3 months, and chronic non-steroidal anti-inflammatory (NSAID) therapy. Additional exclusion criteria included a diagnosis of Crohn's disease (CD), indeterminate colitis, microscopic colitis, ischemic or infectious colitis, human immunodeficiency virus (HIV), or any other immunosuppressive disorder. Participants were required to take precautions against pregnancy during the course of QBECO treatment.

### Tissue collection and pathology scoring

In the UC patient trial, two 2 mm biopsies were taken with each endoscopy from the most inflamed area of colonic mucosa 30–40 cm from the anal verge. If no area of inflammation was identified in the 30–40 cm zone, the biopsies were taken from a randomly chosen site in this zone.

For the experimental models of colitis, murine colon tissue collection was performed as described previously (18). Briefly, mice were anesthetized with isofluorane at the time points indicated, dissected, and had their large bowel collected in 10% neutral buffer formalin for histological analyses or processed for tissue pathology assays. Histology was scored by assessing submucosal edema, crypt hyperplasia, epithelial damage/integrity, and immune cell infiltration. Tissue sections were assessed for (i) submucosal edema (0- no change 1- mild 2- moderate 3- profound), (ii) hyperplasia (0- no change 1−1 to 50%, 2−51 to 100%, 3–>100%), (iii) goblet cell depletion (0 no change 1 mild depletion 2 severe depletion 3- absence of goblet cells), (iv) epithelial damage/integrity (0 no change 1- few cells sloughing, epithelial surface rippled 2- epithelial surface is rippled, damaged 3- epithelial surface is severely disrupted/damage, large amount of cells sloughing). (v) mononuclear cells infiltration (per 40 × field) (0- no change 1– <20, 2−20 to 50, 3–>50 cells). The maximum possible score was 12 For spleen weight, spleen was extracted and weighted.

### Histologic scoring and neutrophil assessment from biopsy samples

Biopsy samples were subjected to H&E staining and subsequently graded using the Geboes' scoring system by a clinical GI pathologist (J.B) (19). Slides were scored twice, double-blinded to both the timing of the biopsy sample and the patient's identity. The Geboes' scoring system classifies histological changes on an ordinal scale: grade 0 (structural change only), grade 1 (chronic inflammation), grade 2 (A. lamina propria neutrophils; B. lamina propria eosinophils), grade 3 (neutrophils in the epithelium), grade 4 (crypt destruction), and grade 5 (erosion or ulcers). An overall score was then generated from 0 to 5.4, with higher scores indicating greater inflammation. The cumulative score has also been converted to a scale (0–22) for its use as a continuous variable (19). Separately, neutrophils were quantified from H&E slides in a blinded manner.

### Statistical analysis

Data was assessed for normal distribution and comparisons were made using non-parametric tests where appropriate. All analyses were performed using two-tailed tests and results are expressed as the mean value ± S.E.M. A *p*-value of ≤0.05 was considered significant.

## Results

### Prophylactic QBECO treatment protects mice from dextran sodium sulfate (DSS)-induced colitis

**Figure 1:**
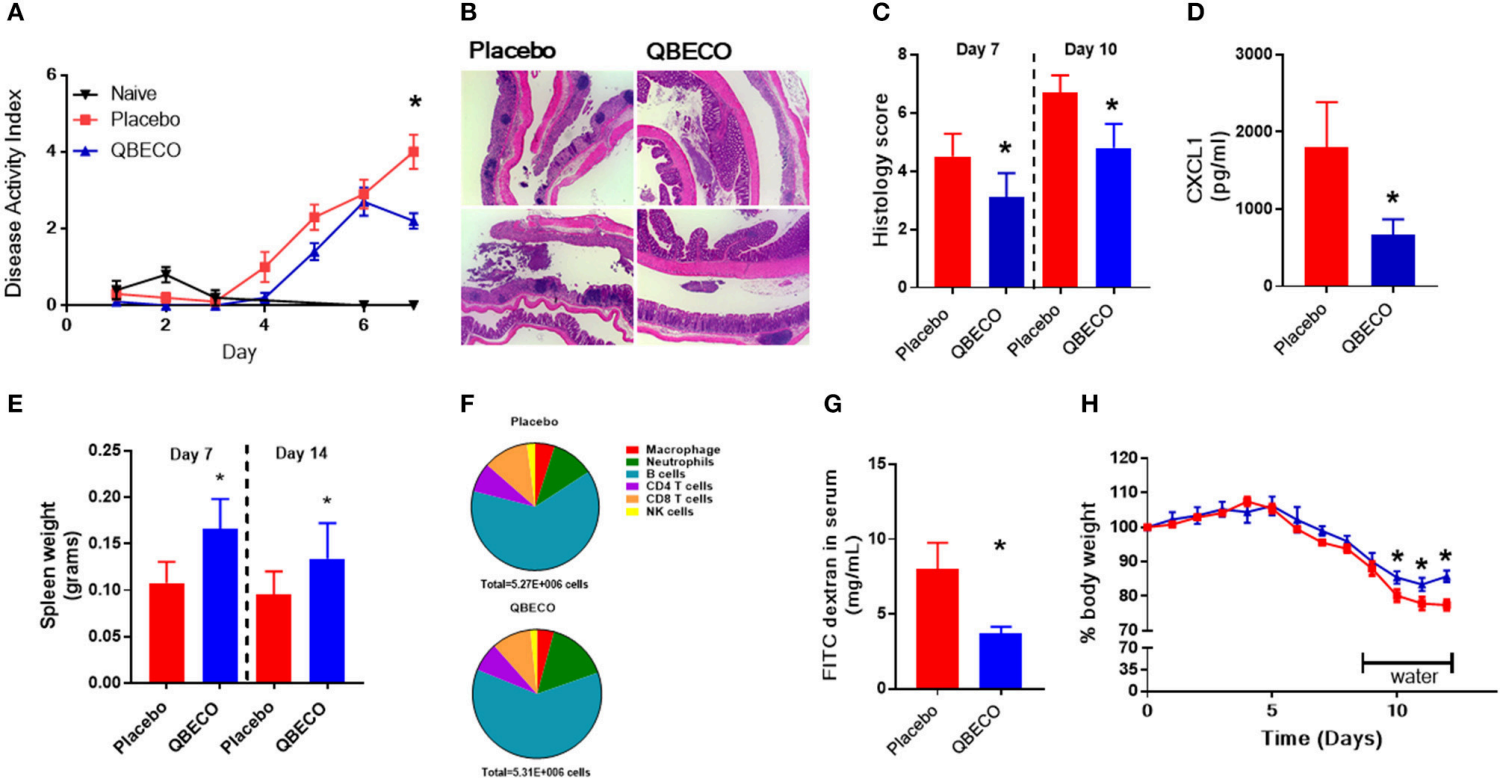
Prophylactic subcutaneous QBECO injections ameliorate severity of DSS induced injury. Mice were prophylacticly treated with placebo or QBECO every other day throughout the course of DSS exposure for 7 days (2.0 or 2.5%). DSS severity was assessed by **(A)** disease severity index, **(B)** histology, **(C)** histological scoring, **(D)** CXCL1 levels, **(E)** spleen weight, **(F)** immune cell profiling in the colon **(G)** barrier function assessed by FITC dextran permeability, and **(H)** recovery rate from 2% DSS. Control *n* = 15 QBECO: *n* = 10, mean ± SEM, *p < 0.05.

The DSS model of colitis was first used to study the potential gastrointestinal protective effects of QBECO. Mice were administered either placebo or QBECO subcutaneously every second day, beginning 10 days prior to and 7 days after DSS administration. Mice treated with QBECO experienced less weight loss as compared to placebo treated mice starting at day 5 following DSS exposure. By Day 7 post-DSS, QBECO treatment had attenuated the DSS-induced weight loss seen in placebo treated mice by >30% (*p* < 0.05). Moreover, the QBECO treated animals displayed a decrease in their disease activity index (placebo: 4.0 ± 1.0 vs. QBECO 2.2 ± 0.4, *p* < 0.05), which collectively accounts for rectal bleeding, weight loss and stool consistency (Figure 1A).

At the histological level, DSS induced substantial damage in the distal colon that included immune cell infiltration, goblet cell depletion, hyperplasia, and loss of epithelial integrity (Figure 1B). In contrast, QBECO treated animals suffered significantly less colonic tissue damage as compared to placebo treated controls at both Day 7 and Day 10 post-DSS (Figures 1B,C). The QBECO treated mice displayed greater epithelial integrity along with fewer infiltrating inflammatory cells in their colonic tissues. Neutrophils comprise the majority of infiltrating cells in this model, and correspondingly, levels of Cxcl1 (a neutrophil chemoattractant also known as keratinocyte chemoattractant [KC]) (placebo: 1798 ± 587 pg/mL vs. QBECO 665 ± 202 pg/mL, *p* < 0.05) were significantly reduced in QBECO treated mice relative to placebo treated DSS controls (Figure 1D). However, systemically, there was clear mobilization and an increase in the number of immune cells in circulation as indicated by the greater spleen weight in QBECO-treated mice (Day 7: 50% increase and Day 14: 40% increase) (Figure 1E); however, the broad distribution of leukocytes was not markedly different in the spleen at Day 14 (Figure 1F) other than slightly more neutrophils. Gastrointestinal barrier function, as assessed by oral fluorescein isothiocyanate dextran (FITC) gavage, was also significantly improved with QBECO treatment, as indicated by reduced levels of FITC-dextran detected in the serum, (Figure 1G; placebo: 8.0 ± 7.1 μg/mL vs. QBECO 3.7 ± 0.5 μg/mL, *p* < 0.05). Finally, QBECO treatment also improved weight loss recovery following DSS exposure when mice were switched back to water (Figure 1H).

### QBECO treatments ameliorate spontaneous colitis in *muc-2*-/- mice

**Figure 2:**
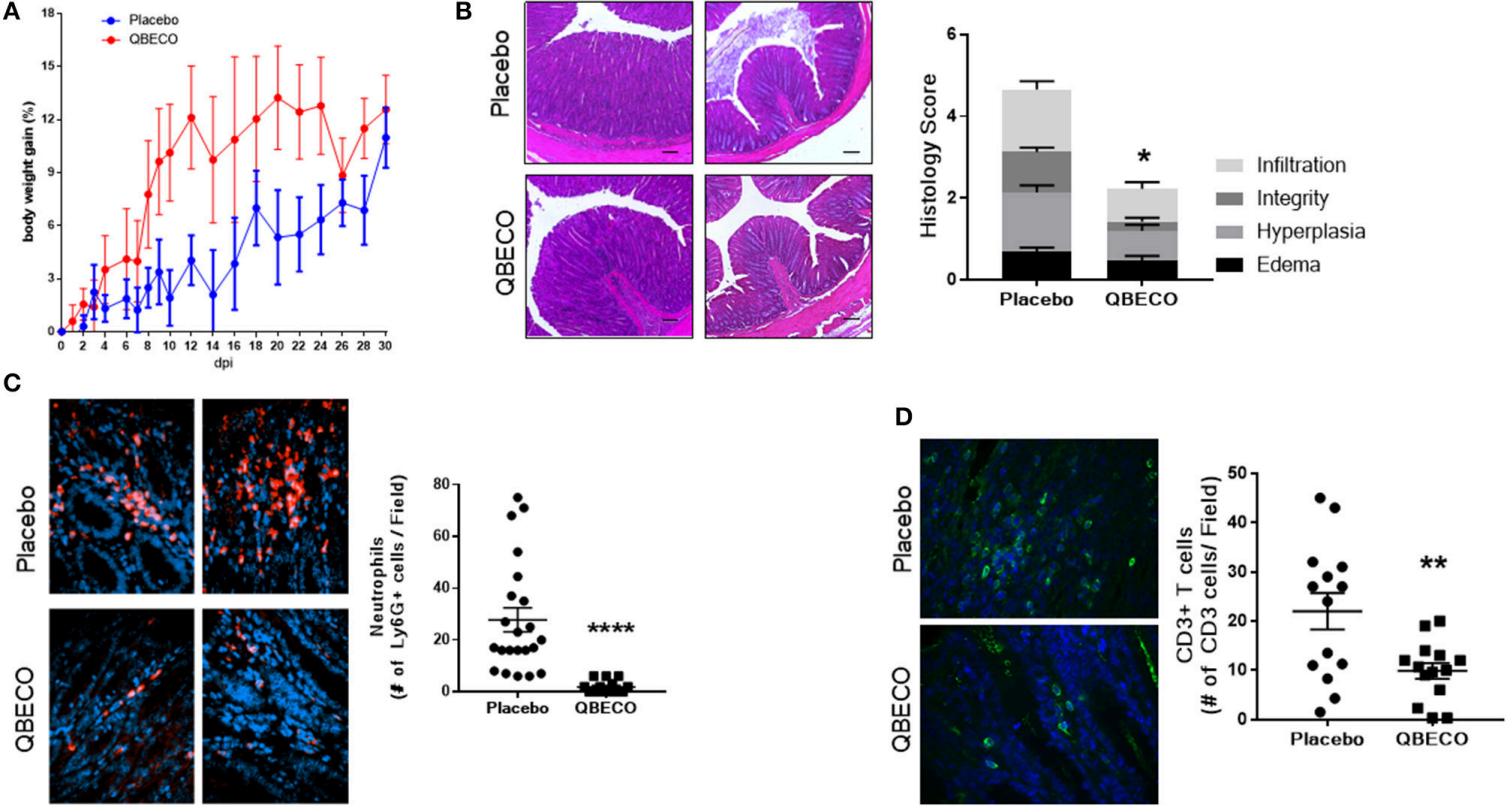
QBECO treatment reduced disease severity in *Muc-2*^−/−^ mice. *Muc2*^−/−^ mice were injected subcutaneously with placebo or QBECO every other day for 30 days and colonic tissue was collected for histology grading **(A,B)**. Colonic tissues were also stained with **(C)** anti-Ly6G to assess neutrophil number and **(D)** anti-CD3 to assess the number of T cells in colonic tissues. Control *n* = 15 QBECO: *n* = 10, mean ± SEM, *p < 0.05; **p < 0.01, ****p = 0.0001.

The *Muc2*^−/−^ model of spontaneous colitis was subsequently used to test the effects of QBECO in a model that better reflects the human disease (20, 21). Three-month old *Muc2*^−/−^ mice (the age when spontaneous colitis overtly develops) (22) were subcutaneously injected with placebo or QBECO every second day for 30 days. As shown in Figure 2A, QBECO treated *Muc2*^−/−^ mice exhibit more weight gain compared to placebo treated mice (Figure 2A). At the end of the 30-day treatment period, widespread infiltration of inflammatory cells, overt crypt hyperplasia, and loss of epithelial integrity were observed in the colons of placebo treated *Muc2*^−/−^ mice; however, these histopathological features were reduced in mice treated with QBECO (Figure 2B). Histopathological scoring (based on inflammatory cell infiltration, hyperplasia, and loss of epithelial integrity) of colonic tissues confirmed greater disease severity (placebo: 4.7 ± 1.5 vs. QBECO 2.2 ± 1.6, *p* < 0.05) in placebo treated mice as compared to QBECO treated animals (Figure 2B). As shown in Figure 2B, QBECO treatment significantly decreased crypt hyperplasia and inflammatory cell infiltration and improved epithelial integrity as compared to placebo control. These data suggest that QBECO treatment administered at the onset of disease markedly attenuates the development of spontaneous colitis in *Muc2*^−/−^ mice.

### QBECO-treatment of *muc2-/-* mice induces selective mucosal immune modulation resulting in the reduction of infiltrating neutrophils and T cells in colonic tissue

**Figure 3:**
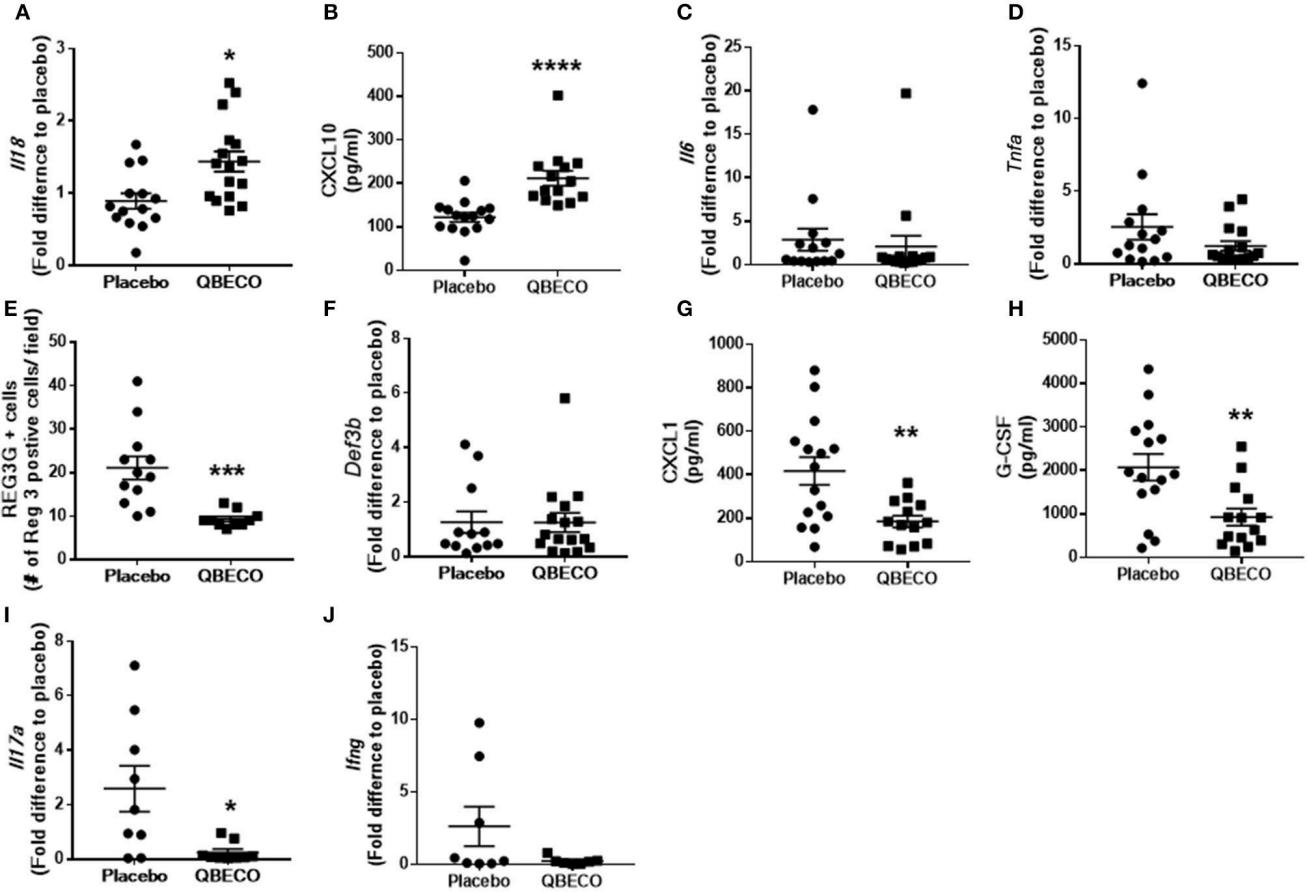
Gene and protein expression of inflammatory molecules were measured in the blood of Muc2^−/−^ mice treated with placebo or QBECO for 30 days. At Day 30, the following immune markers were assessed: **(A)** *Il18*, **(B)** CXCL10, **(C)** *Il6*, **(D)** *Tnfa*, **(E)** REG3G, **(F)** *Def3b*, **(G)** CXCL1, **(H)** G-CSF, **(I)** *Il17a*, and **(J)** *Ifng*. Control *n* = 15, QBECO: *n* = 16, mean ± SEM, *p < 0.05, **p < 0.003, ***p = 0.0001, ****p = 0.0002.

IL-18 plays an important role in intestinal homeostasis, and correspondingly QBECO treatment was found to increase *Il-18* gene transcription (~50% increase) in the colonic tissue of *Muc2*^−/−^ mice along with a concomitant increase in the level of the chemokine Cxcl10 (~70% increase), also known as IFNγ-induced protein 10 (IP-10), in the blood compared to the placebo control (Figures 3A,B). However, no overt changes in the colonic expression of the pro-inflammatory cytokines *Il-6* or *tnfa* were detected (Figures 3C,D). A 50% reduction in the protein levels of Reg3-β, an antimicrobial lectin associated with the development of colitis in *Muc2*^−/−^ (22), was also noted in QBECO treated mice relative to placebo (Figure 3E), suggesting reduced bacterial contact with the colonic mucosal surface, however no changes were observed in β-defensin (another anti-microbial peptide) (Figure 3F). Immunostaining of cellular infiltrates revealed QBECO reduced the number of Ly6G^+^ neutrophils found in the colon to 10% of that of seen in the placebo treated group (Figure 2C). This dramatic reduction in infiltrating neutrophils likely reflected the reduced levels of Cxcl1 and G-CSF detected systemically with QBECO treatment relative to placebo (Figures 3G,H). Similarly, QBECO treated mice showed a 55% reduction in the number of CD3+ T cells in the colonic mucosa relative to placebo (Figure 2D). In corollary, the gene expression or *Il-17A* was markedly attenuated (~90% decrease) in QBECO treated mice relative to those treated with placebo (Figure 3I), with a more moderate reduction in the colonic expression of *Ifng* (Figures 3J), suggesting that QBECO treatment dampens Th17 T cell infiltration and activation - a hallmark of gut pathology in *Muc2*^−/−^ mice (23).

### Tolerability and safety of QBECO treatment in patients with moderate-to-severe ulcerative colitis

Based on the striking beneficial effects of QBECO seen in mouse models, 13 patients with moderate to severe UC were screened for entry into the QBECO open-label study to assess safety, tolerability and efficacy of treatment. Eleven participants, with an average Mayo score at baseline of 8.5 ± 1.5, passed screening. Baseline demographic characteristics are shown in Table 1.

**Table 1:** Demographics of Ulcerative Colitis Study Subjects.

**Characteristic**	
**Age at diagnosis**	**Years**
**Mean**	**29.1**
**Median**	**30.9**
**Range**	**20.1–35.8**
**Age at randomization**	**Years**
**Mean**	**38.5**
**Median**	**38.7**
**Range**	**26.5–53.0**
**Disease duration**	**Years**
**Mean**	**9.4**
**Median**	**7.7**
**Range**	**1.5–20.2**
**Sex**	***n* (%)**
**Male**	**7 (63.6)**
**Female**	**4 (36.4)**
**Baseline disease severity score**	**Mayo score ±*SD***
**Baseline Mayo Score**	**8.5 ± 1.5**


Compliance with treatment administration was high (97% of expected injections) at Week 16. Overall, QBECO was well-tolerated. During the course of the study, 10 of the 11 patients experienced adverse events (AEs; presented in Supplemental Table 1). The most common AEs reported were injection site reactions (*n* = 5; 45%) and transient fatigue (*n* = 4; 36%). One study patient experienced a serious AE during Week 16 of the study when presenting with sepsis-like syndrome (pyrexia, extreme fatigue, tachycardia). Chest X-ray revealed no infiltrates, while computerized tomographic (CT) scans of the abdomen and pelvis were unremarkable, and blood cultures were negative. The condition was considered resolved and the patient was discharged within 2 days without intervention. This patient had been on the lowest dose (0.02 mL) of QBECO, and it was not conclusive whether this episode was related to QBECO treatment or instead a manifestation of UC. As per protocol, the patient had been withdrawn from the study after this episode and categorized as a non-responder, but this subject had initially been observed to be a strong responder to QBECO treatment showing a 6-point drop in full Mayo score from baseline to Week 8 (patient ID 4; Table 2).

**Table 2:** Change in Mayo Score with QBECO Treatment.

**Participant**	**Dose (mL)**	**Baseline Mayo score**	**Week 8 Mayo score**	**Week 16 Mayo score**	**Week 16 status**
1	0.02	11	8	4	Responder
2	0.1	8	3	3	Responder
3	0.05	6	1	2	Remission
4	0.02	9	3	N/A	Non-responder^*^
5	0.05	10	8	5	Responder
6	0.1	7	N/A	8	Non-responder
7	0.1	8	N/A	8	Non-responder
8	0.1	8	N/A	5	Responder
9	0.05	10	N/A	7	Responder
10	0.02	9	N/A	6	Responder
11	0.02	7	N/A	5	Non-responder
Average		8.5 ± 1.5	4.6 ± 3.2	5.3 ± 2.0	

* *Withdrawn due to SAE, N/A, scores not available*.

### Clinical improvement with reduced rectal bleeding observed in patients with ulcerative colitis treated with QBECO

After 16 weeks of QBECO treatment, 63.6% of the subjects (7 out of 11) were deemed to be QBECO responders (i.e., having a decrease in their Mayo score of ≥3 points), with one (9%) achieving remission (Table 2). The one patient who was a responder at Week 8 but who subsequently experienced an SAE (noted previously) did not have a Week 16 endoscopy. Overall Mayo scores dropped from 8.5 ± 1.5 at baseline to 5.3 ± 2.0 at Week 16, with a decrease in severity from baseline to Week 16 for all four sub-components (Supplemental Table 2).

Endoscopic evaluation of disease severity decreased from an average score of 2.2 at baseline to 1.6 at Week 16, and stool frequency decreased from an average score of 2.5 at baseline to 1.6 at Week 16. The most notable and rapid response to QBECO treatment was a marked decrease in rectal bleeding, decreasing from an average score of 1.8 at baseline to 0.6 at Week 16. All subjects had exhibited rectal bleeding at screening, but only 45.5% did so at Week 8. No dose response was observed in this dosing range as improvement was observed in all three randomized dosing groups (0.02, 0.05, or 0.1 mL).

### Patients with ulcerative colitis treated with QBECO had markedly reduced histological damage and lower neutrophil numbers

**Figure 4:**
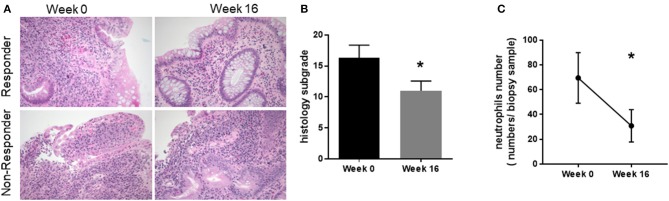
QBECO treatment reduced histological pathology and neutrophil numbers. **(A)** H&E staining was performed on patient biopsy samples collected at Week 0 and Week 16. Responders are defined by a decrease in Mayo score of at least 3 points **(B)** Tissues were scored using Geboe's grading and graphed on a 22- point scale. **(C)** Neutrophils were enumerated from the biopsy samples collected at Week 0 and Week 16. Week 0 *n* = 11, Week 16 *n* = 8, mean ± SEM, *p < 0.05 from paired-*t*-test.

Endoscopic assessment indicated QBECO treatment was associated with reduced macroscopic damage. Histological investigation was undertaken to assess whether QBECO treatment also ameliorated colonic inflammation at a deeper level. Colonic biopsy samples from patients were collected at baseline (Week 0) and at Week 16 of QBECO treatment. Tissues were stained by H&E and examined microscopically for histopathological changes by a clinical pathologist who was blinded to both times of tissue collection (i.e., Week 0 or Week 16) and clinical response to QBECO. Patients receiving QBECO for 16 weeks showed significant improvements in histology compared to baseline (Week 0: 16.3 ± 7.0 vs. Week 16: 11.0 ± 3.8, *p* < 0.05; Figures 4A,B). QBECO treatment was found to reduce the severity of mucosal damage and improved mucosal integrity. Of note, the number of infiltrating neutrophils, one of the hallmarks for acute UC correlated with disease severity (24, 25), was reduced by 50% with QBECO treatment, relative to baseline (Figure 4C).

### QBECO treatment selectively modulates the expression of specific cytokines in the colons of patients with ulcerative colitis

**Figure 5:**
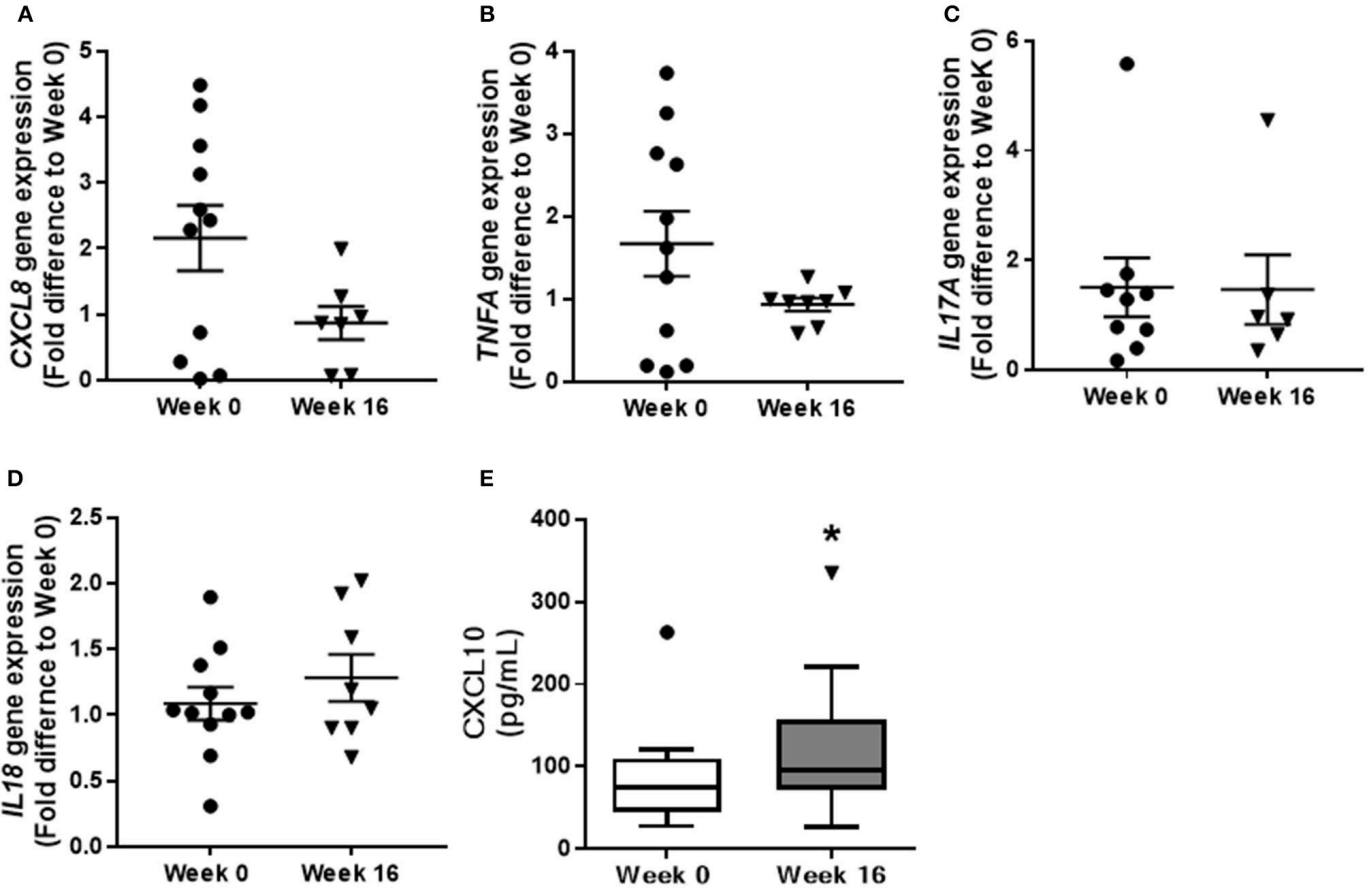
QBECO treatment modulates gene expression in colonic tissue of UC patients. Transcription of inflammatory genes, **(A)** *CXCL8*, **(B)** *TNFA*, **(C)** *IL17A*, and **(D)** *IL18* was examined from biopsy samples collected at Week 0 and Week 16 following QBECO treatment. **(E)** CXCL10 level were measured in the serum of UC patients at Week 0 and Week 16 following QBECO treatments. Week 0 *n* = 11, Week 16 *n* = 8, mean ± SEM, *p < 0.05.

Gene transcription of pro-inflammatory factors was examined in colonic biopsy samples to further characterize the influence of QBECO treatment on colonic inflammation. Lower levels of *CXCL8*, a neutrophil chemoattractant, were observed at Week 16 as compared to baseline (Figure 5A). This reduction in colonic tissue expression of *CXCL8* correlated with the observed reduction in neutrophil infiltration into the colonic mucosa (Figure 4C). There were no marked differences between baseline and Week 16 in the expression of other pro-inflammatory genes such as *IL17A* or *IL18* in colonic tissues (Figures 5B–D); however, there was a trend toward a reduction in the expression of *TNFA*. Similar to what was seen in the experimental models, serum levels of CXCL10 were increased in UC patients with QBECO treatment (Figure 5E).

## Discussion

UC is a chronic disease characterized by severe mucosal inflammation of the large bowel. Although its etiology remains largely undefined, it is thought to be multifactorial - integrating genetic, environmental and immunological factors. Supporting barrier function and limiting damaging inflammatory cell infiltration is thought to be essential to restoring mucosal homeostasis and improving the course of disease progression in UC (10, 26). However, the current treatment paradigm, which is focused on suppression of inflammation, hampers the body's intrinsic ability to properly resolve inflammation (1, 4), since a number of studies have demonstrated that innate immune signaling plays a key role in promoting mucosal homeostasis and healing (27–29). In this study, we demonstrate that by selectively augmenting immune function by administering a gut microbe-based immunotherapy subcutaneously, we were able to reduce gastrointestinal pathology at the histological level in UC patients.

In recent years, genetic association studies have suggested that aberrant mucosal immune responses to gastrointestinal bacteria predispose to the development of IBD (30). Although certain immune defects, such as alterations in the Th17 pathway, appear common to both CD and UC, gene variants associated with UC in particular underscore the role of altered epithelial barrier function in disease development (30). The collective evidence derived from assessing colonic biopsies from both the preclinical and clinical samples show that QBECO treatment potentiates immunological processes that help restore gastrointestinal barrier function and mucosal homeostasis. This is in line with studies showing that activation of pathways involved in Toll-like receptor (TLR) signaling is important in regulating mucosal immune homeostasis and maintaining epithelial barrier function (27, 28). DSS-exposed mice treated with QBECO experienced less weight loss and displayed an overall reduced severity of colitis. Importantly, in this model QBECO treated animals were found to have greater bowel integrity, as demonstrated through reduced permeability to FITC-dextran. In *Muc2*^−/−^ mice that develop intestinal inflammation in a manner more similar to that seen in UC patients (11), QBECO treatment attenuated intestinal pathology, improved mucosal integrity, decreased crypt hyperplasia, resulting in reduced immune cell infiltration of the gastrointestinal mucosa.

The majority of patients treated with QBECO showed clinical, endoscopic and histological improvement of their disease following 16 weeks of treatment. In addition to endoscopic healing, QBECO treatment led to an improvement in histopathology, including reduced mucosal damage and improved mucosal integrity. Although the number of patients treated in this initial study is small, These observations are noteworthy as histologic remission/improvement, which is distinct from the more superficial assessment of endoscopic healing, has been shown to be a better predictor for long-term clinical outcomes with lower rates of corticosteroid use and acute severe colitis over a 6-year period (31). Consistent with the improvements in histology seen with QBECO treatment, the most rapid and dramatic change with QBECO treatment was a reduction in rectal bleeding, observed within 1 week, with further improvement seen over time. Tolerability, compliance and the safety of the treatment regimen was good. The main side effect observed in patients to date has been transient flu-like symptoms, which is expected given the mechanism of action of the QBECO as an immune stimulant. Flu-like symptoms in response to QBECO may in fact be a good sign as it indicates the patient's immune system is responding to treatment.

Neutrophil infiltration in UC contributes to loss of barrier function, epithelial apoptosis and oxidative tissue damage (24, 25). QBECO treatment reduced the number of neutrophils in the colonic mucosa in both UC patients and in our experimental models of colitis. Congruent with the observed reduction in neutrophil infiltration in colonic tissue, QBECO treatment led to a reduction in the levels of neutrophil chemoattractants. This may seem counter-intuitive given QBECO activates innate immune responses; however, neutrophil recruitment to the gut in UC is thought to result from poor barrier function resulting in microbial encroachment and invasion of the mucosal epithelium (24). Given the evidence that QBECO treatment improves bowel integrity, it is expected that there be a reduced need to recruit neutrophils to clear bacteria from the mucosal surface. However, systemically, CXCL10 levels were increased with QBECO treatment in both experimental colitis and in UC patients. CXCL10 expression is turned on by IFNγ signaling (32), which we know through *in vitro* stimulation studies using human leukocytes, is enhanced by QBECO administration (unpublished data). This is of note because IFNγ has been shown to be important for regulating epithelial homeostasis and dampening colonic inflammation (33, 34). In both animal and human studies, the consequence of repeated immune activation with QBECO treatment has not shown to be associated with concerning immune-related toxicity; however, larger trials with a longer duration of follow-up are needed to confirm safety.

In conclusion, QBECO treatment, which induced both endoscopic healing and histologic improvement, shows promise as an intriguing novel therapeutic approach for the treatment of UC. Unlike current UC treatments focused on managing symptoms via immune suppression, these findings suggest that immune stimulation by QBECO treatment enhances immune competency in the gastrointestinal tract thereby addressing the defective barrier function in the colon. The histopathology and biomarker analyses performed in UC patients treated with QBECO and in our experimental models of colitis provide insight into the biologic pathways involved in QBECO efficacy; these will be useful in dissecting the molecular mechanisms through which this microbial-based treatment works to improve colonic barrier function and integrity. These foundational results are encouraging, and larger follow-on trials are now needed to confirm these findings and to demonstrate QBECO treatment can sustain disease remission in patients living with UC.

## Ethics statement

This study was carried out in accordance with the recommendations of Canadian Council of Animal Care. The protocols were approved by the animal care committees at the University of British Columbia and Qu Biologics Inc.

## Author contributions

HS and SK interpreted the results and wrote the first draft of the manuscript. HS, MaB, and MoB managed the preclinical studies, performed the experiments, and conducted the analyses. HY, BL, HL, VM, and HBY helped perform the experiments. JP, SS, BB, JM, RF, JC, MJ, and HG contributed to clinical study design and execution. SK and BV designed the experimental study plan. All authors reviewed and provided feedback on the final version of the manuscript.

### Conflict of interest statement

HS, MaB, MoB, BL, JP, MJ, JC, and SK are (or were when the study was conducted) employees of Qu Biologics, a clinical-stage biotechnology company. HG is co-founder and CEO of Qu Biologics. SS is the Chief Medical Officer and member of the Board of Directors of Qu Biologics. BB (has Qu share options), JM, and BV have functioned as clinical and academic investigators on Qu Biologics sponsored studies. The remaining authors declare that the research was conducted in the absence of any commercial or financial relationships that could be construed as a potential conflict of interest.
