# Supplementary material for: Immune Stimulation Using a Gut Microbe-Based Immunotherapy Reduces Disease Pathology and Improves Barrier Function in Ulcerative Colitis
Source: Front Immunol. 2018 Sep 27;9:2211. doi: 10.3389/fimmu.2018.02211 (PMC6170651; doi:10.3389/fimmu.2018.02211)
Supplement: Supplementary file 1 [file Data_Sheet_1.docx]

**Supplemental Table 1: Summary of all Treatment Emergent adverse events, subjects’ maximum severity by MedDRA SOC and PT**

|  | **All Timepoints (N = 11) __________________** | | |
| --- | --- | --- | --- |
| **System Organ Class        Preferred Term, n (%)** | **Mild** | **Mod** | **Sev** |
| **Any Adverse Event** | **2 (18.2)** | **6 (54.5)** | **2 (18.2)** |
| **Blood and lymphatic system disorders** | **0 (0)** | **1 (9.1)** | **0 (0)** |
| *Iron deficiency anemia* | 0 (0) | 1 (9.1) | 0 (0) |
| **Eye disorders** | **0 (0)** | **1 (9.1)** | **0 (0)** |
| *Iritis* | 0 (0) | 1 (9.1) | 0 (0) |
| **Gastrointestinal disorders** | **0 (0)** | **3 (27.3)** | **1 (9.1)** |
| *Abdominal distension* | 1 (9.1) | 0 (0) | 0 (0) |
| *Diarrhea* | 0 (0) | 1 (9.1) | 0 (0) |
| *Gastrointestinal motility disorder* | 1 (9.1) | 0 (0) | 0 (0) |
| *Nausea* | 1 (9.1) | 2 (18.2) | 0 (0) |
| *Pancreatitis acute* | 0 (0) | 0 (0) | 1 (9.1) |
| *Vomiting* | 3 (27.3) | 0 (0) | 0 (0) |
| **General disorders and administration site conditions** | **4 (36.4)** | **3 (27.3)** | **1 (9.1)** |
| *Fatigue* | 0 (0) | 3 (27.3) | 1 (9.1) |
| *Influenza like illness* | 0 (0) | 1 (9.1) | 0 (0) |
| *Injection site bruising* | 1 (9.1) | 0 (0) | 0 (0) |
| *Injection site pruritus* | 3 (27.3) | 0 (0) | 0 (0) |
| *Peripheral swelling* | 1 (9.1) | 0 (0) | 0 (0) |
| *Pyrexia* | 0 (0) | 0 (0) | 1 (9.1) |
| *Vaccination site hematoma* | 1 (9.1) | 0 (0) | 0 (0) |
| **Infections and infestations** | **1 (9.1)** | **3 (27.3)** | **0 (0)** |
| *Gastroenteritis* | 0 (0) | 2 (18.2) | 0 (0) |
| *Oral herpes* | 1 (9.1) | 0 (0) | 0 (0) |
| *Viral upper respiratory tract infection* | 1 (9.1) | 1 (9.1) | 0 (0) |
| **Injury, poisoning and procedural complications** | **0 (0)** | **1 (9.1)** | **0 (0)** |
| *Back injury* | 0 (0) | 1 (9.1) | 0 (0) |
| **Metabolism and nutrition disorders** | **0 (0)** | **1 (9.1)** | **0 (0)** |
| *Dehydration* | 0 (0) | 1 (9.1) | 0 (0) |
| **Nervous system disorders** | **1 (9.1)** | **1 (9.1)** | **0 (0)** |
| *Dysgeusia* | 1 (9.1) | 0 (0) | 0 (0) |
| *Headache* | 1 (9.1) | 1 (9.1) | 0 (0) |
| **Psychiatric disorders** | **0 (0)** | **1 (9.1)** | **0 (0)** |
| *Anxiety* | 0 (0) | 1 (9.1) | 0 (0) |
| *Insomnia* | 0 (0) | 1 (9.1) | 0 (0) |
| **Respiratory, thoracic and mediastinal disorders** | **1 (9.1)** | **0 (0)** | **0 (0)** |
| *Oropharyngeal pain* | 1 (9.1) | 0 (0) | 0 (0) |

**Supplemental Table 2: Component-wise analysis of Mayo scores**

|  |  | **Baseline** |  | **Week 8** | |  | **Week 16** |  |
| --- | --- | --- | --- | --- | --- | --- | --- | --- |
| Stool Frequency | Normal | 0 (0%) |  | 0 (0%) |  | | 1 (9.1%) |  |
|  | 1-2/day more than normal | 3 (27.2%) |  | 6 (54.5%) |  | | 4 (36.4%) |  |
|  | 3-4/day more than normal | 0 (0%) |  | 1 (9.1%) |  | | 4 (36.4%) |  |
|  | >4/day more than normal | 8 (81.8%) |  | 4 (36.4%) |  | | 2 (18.2%) |  |
| Rectal Bleeding | None | 0 (0%) |  | 6 (54.5%) |  | | 8 (72.7%) |  |
|  | Visible Blood with stool < ½ time | 4 (36.4%) |  | 3 (27.3%) |  | | 1 (9.1%) |  |
|  | Visible Blood with stool ≥ ½ time | 5 (45.5%) |  | 2 (18.2%) |  | | 1 (9.1%) |  |
|  | Passing blood alone | 2 (18.2%) |  | 0 (0%) |  | | 1 (9.1%) |  |
| Mucosal Appearance at Endoscopy | Normal | 0 (0%) |  | 1 (20%) |  | | 1 (10%) |  |
|  | Mild disease | 0 (0%) |  | 2 (40%) |  | | 3 (30%) |  |
|  | Moderate disease | 9 (81.8%) |  | 0 (0%) |  | | 5 (50%) |  |
|  | Severe disease | 2 (18.2%) |  | 2 (40%) |  | | 1 (10%) |  |
| Physician Rating of Disease Activity | Normal | 0 (0%) |  | 1 (12.5%) |  | | 0 (0%) |  |
|  | Mild | 1 (9.1%) |  | 3 (37.5%) |  | | 6 (54.5%) |  |
|  | Moderate | 9 (81.8%) |  | 4 (50%) |  | | 4 (36.4%) |  |
|  | Severe | 1 (9.1%) |  | 0 (0%) |  | | 1 (9.1%) |  |
| *****Not all subjects received an endoscopy at all timepoints. Percents within each category are based on subjects that have a value for that sub-score at the given timepoint. | | | | | | | | |
